# Advancing Understanding of Cerebrovascular Hemodynamic Perturbations in Pediatric Cerebral Malaria Using a Modified Critical Closing Pressure Evaluation- A Prospective, Observational Study

**DOI:** 10.1007/s12028-025-02245-w

**Published:** 2025-04-21

**Authors:** Nicole F. O’Brien, Madiha Q. Raees, Hunter J. Wynkoop, Mengxin Yu, Dylan Small, Karl B. Seydel, Montfort Bernard Gushu, Tusekile Phiri, Sylvester June, Terrie E. Taylor

**Affiliations:** 1 Department of Pediatrics, Division of Critical Care Medicine, Nationwide Children’s Hospital, The Ohio State University, 700 Children’s Drive, Columbus, OH 43502 USA; 2 Department of Anesthesiology and Critical Care Medicine, Division of Critical Care Medicine, The Children’s Hospital of Philadelphia, University of Pennsylvania Perelman School of Medicine, 3401 Civic Center Blvd, Rm 9NW103, Philadelphia, PA 19104 USA; 3 Department of Statistics and Data Science, The Wharton School, University of Pennsylvania, 3733 Spruce St, Philadelphia, PA 19104 USA; 4 The Wharton School, University of Pennsylvania, 3733 Spruce St, Philadelphia, PA 19104 USA; 5 Department of Osteopathic Medical Specialties, College of Osteopathic Medicine, Michigan State University, 909 Wilson Road, B309-B W. Fee Hall, East Lansing, MI 48824 USA; 6 The Blantyre Malaria Project, Queen Elizabeth Central Hospital, Private Bag 360, Chichiri, Blantyre 3, Malawi

**Keywords:** Cerebral malaria, Transcranial doppler ultrasound, TCD, Critical closing pressure, CrCP, Diastolic closing margin, Optimal cerebral perfusion pressure

## Abstract

**Background:**

Cerebral malaria (CM) results in significant mortality globally. Abnormal cerebral blood flow (CBF) has been described in CM and may contribute to poor outcomes. Changes to vascular tone may be contributing to flow aberrations but measuring it in the clinical setting is difficult. Critical closing pressure (CrCP) is calculated as CrCP = intracranial pressure (ICP) + vascular tone + venous pressure. If CrCPs other components are determined, vascular tone can be inferred. CrCP can also be used to determine the diastolic closing margin (DCM = diastolic blood pressure (DBP)-CrCP) which represents the lower safety limit of cerebral perfusion pressure.

**Methods:**

Children 6 months-12 years with CM and age-matched healthy controls were enrolled. Using concurrent transcranial doppler ultrasound (TCD) CBF velocities and systemic blood pressure measurements, CrCP was determined, and DCM calculated. Non-invasive estimates of ICP were assessed and venous flow was measured. Vascular tone was deduced. Differences in CrCP between controls and CM patients were determined. DCM and its association with outcome was assessed.

**Results:**

We enrolled 220 children with CM and 400 controls. In CM patients, there were significantly more children with CrCP > 1SD below (n = 37, 17%) and > 1 SD above (n = 42, 19%) the mean normal value of the control group (n = 15, 5% > 1SD below and n = 20, 5% > 1 SD above, *p* < 0.001 for both). Opening pressure, an estimate of ICP, was not different between patients and controls. Venous flows were higher in children with CM than controls, but no difference was seen in CM patients with CrCP less than, within, or greater than 1SD from normal. A DCM < 20mmHg conferred a relative risk of poor outcome (RR 1.4, 95%CI 1.2–1.9, *p* = 0.008).

**Conclusions:**

CrCP was > 1SD lower or higher than the mean normal value in a significant number of children with CM. A low DCM is associated with a worse prognosis and may serve as a therapeutic target.

**Supplementary Information:**

The online version contains supplementary material available at 10.1007/s12028-025-02245-w.

## Background

Malaria affects 240 million individuals and results in ~ 620,000 deaths annually, > 80% of which occur in African children [1]. That equates to > 1300 childhood deaths daily. Cerebral malaria (CM) is a severe form of the disease characterized by deep coma not attributable to other causes. Even with effective antimalarial drugs, CM results in case fatality rates of 15–30% [2]. Among survivors, 30–50% are left with long-term neurologic complications including motor deficits, cognitive and behavioral issues, and epilepsy [3]. More than 30 randomized clinical trials of adjunctive therapies have failed to improve the poor outcomes in CM, likely due to an incomplete understanding of the neuropathogenesis of the disease. To improve this understanding, the cerebrovascular hemodynamics of two populations of African children with CM have been assessed using transcranial doppler ultrasound (TCD) [4, 5]. In both cohorts, based on distinct alterations to measured cerebral blood flow velocities (CBFVs) and morphologic waveforms, five different phenotypes were described.

Cerebral blood flow (CBF) is determined by the cerebral perfusion pressure (CPP) divided by the cerebrovascular resistance (CVR) or vascular tone. CPP is determined as the difference between the mean arterial pressure (MAP) and intracranial pressure (ICP). Therefore, CBF = (MAP-ICP)/CVR [7]. Alterations to any of these factors result in changes to CBF and measured TCD CBFVs and waveform morphology. Having previously established that mean blood pressures and estimates of ICP were not different between TCD phenotypic groups, we hypothesize that perturbation of CVR/vascular tone is the primary contributor to abnormal TCD findings in children with CM [5]. Because vascular tone is difficult to measure directly in the clinical setting, we used an alternate approach to infer it and test our primary hypothesis.

The critical closing pressure (CrCP) is defined as the absolute driving pressure below which collapse of the cerebral circulation occurs and blood flow ceases. As such, CrCP accounts for the cumulative effect of all downstream factors resisting forward CBF and is calculated as: CrCP = tissue pressure (ICP) + vascular tone + venous pressure [8–13]. CrCP can be assessed non-invasively using TCD by creating a linear regression line of time averaged systolic and diastolic flow velocities and simultaneously acquired systolic and diastolic blood pressures (DBP) [14]. Beyond gaining insight into the cerebrovascular hemodynamics,, CrCP may also be a valuable clinical tool. It can be used to calculate the diastolic closing margin (DCM = DBP-CrCP), which allows cerebral perfusion pressure to be optimized to ensure forward cerebral blood flow, decreasing the risk of cerebral ischemia and secondary brain injury. Thus, a secondary objective of our study was to explore the DCM in children with CM and evaluate its relationship to outcomes.

## Materials and Methods

### Patient Population and General Care of CM Patients

This study occurred at Queen Elizabeth Central Hospital/Pediatrics Research Ward in Blantyre, Malawi from January 2019-June 2023. Parents or guardians of all children 6 months to 12 years of age who met the World Health Organization case definition of cerebral malaria (*Plasmodium falciparum* parasitemia, Blantyre Coma Score (BCS) ≤ 2, and no other discernable cause of encephalopathy) were approached for enrollment. The BCS was developed in 1987 in Blantyre, Malawi to assess a child’s level of consciousness and prognosticate outcomes during severe falciparum malaria. A common feature in children with cerebral malaria is that the eyes are spontaneously open despite a deep level of coma, making other traditional scoring tools such as the Glasgow Coma Scale less helpful in this population. Therefore, the BCS is based on directed eye movements rather than spontaneous eye opening and also includes the verbal and motor response of the child. The total score ranges from 0 to 5 (0 is the worse, 5 is the best).

Children with sickle cell disease (known or suspected) were excluded, given the high frequency of abnormal TCD examinations in this population. Likewise, given the unknown impact of severe malnutrition (mid-upper arm circumference < 11 cm) or advanced HIV disease (known HIV positive status with severe wasting) on TCD examinations, these children also were excluded. Lastly, if TCD was attempted but no acoustic window to allow a technically adequate examination could be identified, these participants were excluded.

Demographic data, vital signs, and physical examination findings were collected. Finger-prick samples were analyzed to determine parasite species and density, the-packed cell volume (PCV), and blood glucose and lactate concentrations (Aviva Accu-Check, Zurich, Switzerland and Arkray Lactate Pro 2, Kyoto, Japan). Finger-prick samples were also obtained for blood gas analysis (Abbot iSTAT, Chicago, Illinois, USA). Venous blood was taken to determine the total body parasite biomass, measured by quantifying Plasmodium falciparum histidine-rich protein 2 (PfHRP2). An admission lumbar puncture in the lateral decubitus position was performed, opening pressure (OP) measured, and the cerebrospinal fluid was analyzed for cell counts and chemistry.

All patients underwent an admission electroencephalogram (EEG)(Ceegraph digital machine, BioLogic, Natus Medical Incorporated, Pleasanton, California, USA) with a modified 10–20 system to evaluate for non-convulsive status epilepticus. Duration of EEG was limited to 30 min. EEGs were clinically reviewed by a neurologist with fellowship training in EEG. Participants also underwent a brain MRI (0.064-T Hyperfine Swoop® Guilford, Connecticut, USA) to evaluate the brain volume score (BVS). BVS values of 1–2 represent marked and mild atrophy, respectively. A BVS of 3 represents a normal brain volume whereas scores of 4–8 signify progressive increases in brain volume (BVS 4 = mildly increased brain volume but with normal cisternae, ventricles, and sulci; BVS 5 = mildly increased brain volume but with some localized or regional loss of cisternae, ventricles, and sulci; BVS 6 = moderately increased brain volume with diffuse involvement but incomplete effacement of the cisternae, ventricles, and sulci; BVS 7 = severely increased brain volume with complete cisternal and sulcal effacement but without herniation; BVS 8 = severely increased brain volume with complete cisternal and sulcal effacement with herniation) [15–17]. MRIs were systematically reviewed by radiologists experienced in radiographic findings of children with CM. EEG and MRI were performed within four hours of hospital admission.

All patients received intravenous artesunate according to Malawian national guidelines. Patients received 20 mL/kg of whole blood if admission PCV was < 15% or > 15% but with signs of intolerance (defined as respiratory distress or hemodynamic compromise with capillary refill time > 2 s, weak pulse, and/or cool extremities). Intravenous dextrose (1 mL/kg of Dextrose 50%) was given when documented hypoglycemia occurred (< 3 mmol/L). Clinical seizure activity was treated with 0.2 mg/kg of diazepam followed by phenobarbital 20 mg/kg if it persisted.

### TCD Examination

TCD was performed using a commercially available unit (NovaSignal, Los Angeles, California, USA). The initial TCD examination occurred within 4 h of admission for CM patients. Middle cerebral arteries (MCAs), extracranial internal carotid arteries (Ex-ICA), and basilar arteries were insonated at 2-mm intervals using previously described methods [18]. Systolic (Vs), diastolic (Vd), and mean flow (Vm) velocities were recorded at each interval [19, 20]. In order to estimate the venous component to calculated CrCP, mean blood flow velocity in the deep middle cerebral vein was acquired through the posterior temporal window. [21] Children with admission TCD consistent with terminal intracranial hypertension (tICH) were excluded from analysis since measurement of CrCP is not possible with these TCD findings. Mean blood pressure was obtained simultaneously with the TCD using an automated pediatric blood pressure cuff (Contec, Melbourne, FL).

### CrCP and Diastolic Closing Margin (DCM) Calculations

The average systolic (Vs) and diastolic (Vd) CBFV measured from 18 cardiac cycles (three separate measurements of 6 cycles each) and the simultaneously obtained average systolic (SBP) and diastolic blood pressures (DBP) from three separate checks were utilized [14]. The relationship between Vs, Vd and SBP, DBP were determined by performing linear regression analysis. The intercept of the regression line at the x axis was determined to be the CrCP (Supplementary Fig. 1).

The diastolic closing margin (DCM) represents the minimum pressure required during diastole to avoid cessation of cerebral blood flow during that phase of the cardiac cycle and was calculated as: DCM = DBP-CrCP for all participants [22]. “Low DCM” was defined as a DCM < 20 mmHg. “Critical DCM” was defined as a DCM < 10 mmHg. [22]

### Outcomes

The Pediatric Cerebral Performance Category (PCPC) scoring system is a tool that was developed to measure and quantify morbidity after pediatric critical illness [23, 24]. Scores range from 1 to 6, with 1 being a normal functional level and 6 being death. Other values represent progressive impairment: 2 = mild disability (alert and able to interact at an age appropriate level but with mild cognitive, behavioral, or neurological deficits), 3 = moderate disability (alert and able to carry out age appropriate activities of daily life but with obvious cognitive or neurological deficits that limit function), 4 = severe disability (conscious but dependent on others for all daily functions), and 5 = vegetative state (any degree of coma or an inability to interact with the environment). PCPC was scored at the time of hospital discharge directly by research staff. Children with a PCPC of 1 or 2 were considered to have a good outcome while those with a PCPC of 3 to 5 were considered to have survived with moderate to severe disability. Participants who died were scored as a 6.

### Patient Population- Healthy Controls

Healthy children aged 6 months-12 years were recruited. Children with known or suspected sickle cell disease, severe malnutrition, or advanced HIV disease as defined above were excluded. Children were screened for fever, clinical signs of malarial infection, respiratory infection, and gastroenteritis. If any abnormalities were identified, these children were excluded. Control children underwent routine vital sign measurement to obtain temperature, blood pressure, and oxygen saturation. They also underwent fingerprick to obtain drops of blood to evaluate blood gas (pH, PCO2) and PCV. Healthy control children did not undergo lumbar puncture to obtain opening pressure, nor MRI to assess brain volume, so normative values for these factors were taken from the pre-existing literature [25, 26]. Healthy participants also underwent TCD and CrCP/DCM was calculated in an identical fashion to children with CM.

### Statistics

Categorical values were presented as frequencies with percentages. Other demographics, vital signs, laboratory variables, and CrCP values were tested for normality using the D’Agostino and Pearson as well as the Shapiro–Wilk normality tests and then reported as medians with interquartile ranges or means with standard deviations where appropriate. The relationship between the calculated CrCP of control patients versus those with CM by age as well as the variables that impact CrCP were explored between groups using students t tests or one way analysis of variance where appropriate. Using the robust regression and outliers removal (ROUT) method, no outliers (defined as more than 3SD away from the mean) of CrCP were identified in the dataset, therefore no values were trimmed prior to this analysis [27]. Outcomes were defined as normal, survived but with moderate to severe disability, and died; chi-squared tests were used to evaluate the differences in outcome between CrCP and DCM groups. All analyses were conducted using GraphPad Prism.

## Results

A total of 227 children with CM were screened for inclusion. Four children had no acoustic window to allow for a technically adequate TCD examination, so were excluded. Three children had evidence of terminal intracranial hypertension on initial screening TCD examination and were also excluded. A total of 220 total children with CM underwent all study related procedures. Patient demographics are in Table 1. Four hundred healthy children were enrolled as control patients.

**Table 1:** Cohort demographics (n = 220)

Variable	Value
Demographics	
Age (months), mean (SD)	58 (± 33)
Male, n (%)	114 (52)
Vital signs	
Temperature (°C), mean (SD)	37.0 (± 0.6)
Heart rate (beats/min), mean (SD)	140 (± 12)
RR (breaths/min), mean (SD)	30 (± 5)
Oxygen saturation (%), mean (SD)	98 (± 2)
SBP (mmHg), mean (SD)	100 (± 8)
DBP (mmHg), mean (SD)	59 (± 5)
Laboratory investigations	
Packed Cell Volume (%), mean (SD)	24 (± 3)
Glucose (mmol/L), mean (SD)	5.7 (± 0.9)
Lactate (mmol/L), mean (SD)	2.7 (± 1.2)
Parasites/microliter blood, median [IQR]	255,000 [9100,664000]
PfHRP2 (ng/mL), median [IQR]	571 [215,1335]
Clinical Features	
Blantyre coma score, n (%)	
0	33 (15%)
1	84 (38%)
2	103 (47%)
Outcome	
Good, n (%)	
PCPC 1–2 (normal, mild disability)	147 (67%)
Poor, n (%)	
PCPC 3–5 (moderate to severe disability)	49 (22%)
PCPC 6 (died)	24 (11%)

n = Number, SD = Standard deviations, IQR = Interquartile range, C = Celsius, min = Minute, RR = Respiratory rate, SBP = Systolic blood pressure, DBP = Diastolic blood pressure, MBP = Mean blood pressure, *Pf*HRP2 = Plasmodium falciparum histidine rich protein 2, PCPC = Pediatric cerebral performance category

### CrCP

The mean CrCP for the entire cohort of control patients was 31 ± 4 whereas it was 31 ± 15 for those with CM (Table 2, *p* = 0.78). There were significant differences in the CrCP of control patients when considering age (Table 2, *p* < 0.001), so we further evaluated differences in CrCP between control patients and CM patients by age groups and found no significant differences (Table 2). However, in all age groups, there were significantly more children with CM with a CrCP > 1SD below the mean normative value compared to control patients (37 ((17%)) vs 15 ((4%)), *p* < 0.001)(Table 3). There were also significantly more children with CM with a CrCP > 1SD above the mean normative value compared to control patients (42 ((19%)) vs 20 ((5%)), *p* < 0.001)(Table 3).

**Table 2:** Calculated critical closing pressure by age in healthy controls compared to children with cerebral malaria (mean ± SD)

Age	Control patients CrCP	CM patients CrCP	*P* value†
2–2.9 years	28 ± 5	29 ± 15	0.89
3–4.9 years	29 ± 4	33 ± 17	0.24
5–6.9 years	34 ± 3	33 ± 13	0.54
7–8.9 years	35 ± 6	32 ± 18	0.38
> 9 years	32 ± 6	29 ± 17	0.53
Entire Cohort	31 ± 4	31 ± 15	0.78

SD = Standard deviation; n = Number; CrCP = Critical closing pressure; CM = Cerebral malaria

†p value considered significant if < 0.05

**Table 3:** Frequency of critical closing pressure more than 1 SD below or above the mean for age in both healthy controls and children with cerebral malaria

Age	# of controls with CrCP > 1SD below mean	# of CM patients with CrCP > 1SD below mean	*P* value^⁕^	# of controls with CrCP > 1SD above mean	# of CM patients with CrCP > 1SD above mean	*P* value^⁕^	Total control patients with CrCP > 1SD outside mean	Total CM patients with CrCP > 1SD outside mean	*P* value^⁕^
2–2.9 years Controls n = 80 CM patients n = 60	3 (8%)	12 (20%)	0.006	5 (6%)	12 (20%)	0.03	8 (10%)	24 (40%)	0.001
3–4.9 years Controls n = 100 CM patients n = 68	5 (5%)	10 (15%)	0.04	7 (7%)	16 (24%)	0.008	12 (12%)	26 (38%)	0.001
5–6.9 years Controls n = 100 CM patients n = 42	3 (3%)	6 (14%)	0.02	4 (4%)	6 (14%)	0.04	7 (7%)	12 (28%)	0.003
7–8.9 years Controls n = 60 CM patients n = 27	3 (5%)	5 (19%)	0.07	2 (3%)	5 (19%)	0.03	5 (8%)	10 (38%)	0.008
> 9 years Controls n = 60 CM patients n = 23	1 (2%)	4 (17%)	0.01	2 (3%)	3 (13%)	0.`12	4 (7%)	7 (30%)	0.02
Entire Cohort	15 (4%)	37 (17%)	< 0.001	20 (5%)	42 (19%)	< 0.001	36 (9%)	79 (36%)	< 0.001

SD = Standard deviation; n = number; CrCP = Critical closing pressure; CM = Cerebral malaria

·*p* value considered significant if < 0.05

#### ICP

Tissue pressure (ICP) is a key variable that contributes to CrCP. Two indirect estimates of intracranial pathology/pressure, the volume of brain swelling on magnetic resonance imaging (or the brain volume score ((BVS)) and the opening pressure (OP) on lumbar puncture were measured on all patients with CM at the time of admission concurrently to TCD and blood pressure measurements done to calculate CrCP. The median BVS for the cohort was 5 (IQR 5–6), significantly higher than the normal BVS of 3 (Table 4). There were no significant differences in BVS identified between children with CM and CrCP within 1SD from normal compared to those with CrCP > 1SD below the mean normal (5 (IQR 5, 6)) or > 1SD above the mean normal (6 (IQR 4–7)) (*p* = 0.39)(Table 4).

**Table 4:** Variables of interest between control patients and those with cerebral malaria as well as between subgroups of cerebral malaria patients

Variable	Healthy Controls (n = 400)	All CM patients (n = 220)	*P* value†	CM and CrCP > 1SD below normal (n = 37)	CM and CrCP > 1SD above normal (n = 42)	CM and CrCP ± 1SD from normal (n = 141)	*P* value†*
Opening pressure (cm H20), mean (SD)	18 ± 10	18 ± 8	0.9	18 ± 6	17 ± 7	19 ± 1	0.62
Brain volume score, median [IQR]	3 [3, 3]	5 [5, 6]	0.02	5 [5, 6]	6 [4, 7]	5 [5, 6]	0.39
pH, mean (SD)	7.4 ± 0.03	7.39 ± 0.06	0.9	7.39 ± 0.09	7.4 ± 0.07	7.39 ± 0.07	0.74
CO2 (mmHg), mean (SD)	38 ± 3	27 ± 10	0.04	27 ± 11	24 ± 11	29 ± 8	0.35
Temperature (°C), mean (SD)	36.6 ± 0.3	37.2 ± 0.6	0.67	37.4 ± 0.4	37.4 ± 0.2	37.0 ± 0.2[37.4, 39.1]	0.99
Mean blood pressure (mmHg), mean (SD)	75 ± 8	74 ± 8	0.76	73 ± 6	75 ± 4	74 ± 3	0.99
Oxygen saturation (%), mean (SD)	98 ± 1	98 ± 2	0.99	97 ± 2	98 ± 2	98 ± 1	0.99
Packed Cell Volume (%), mean (SD)	35 ± 4	24 ± 3	0.01	24 ± 3	24 ± 4	24 ± 3	0.99
Seizures on EEG, n (%)	–	0 (0%)	N/A	0 (0%)	0 (0%)	0 (0%)	N/A
Venous flow on TCD, mean (SD)	17 ± 1	28 ± 11	< 0.001	28 ± 10	28 ± 11	29 ± 9	0.16

CM = Cerebral malaria; CrCP = Critical closing pressure; n = Number; cm H20 = Centimeters of water; CO2 = Carbon dioxide; EEG = Electroencephalogram; TCD = Transcranial doppler ultrasound; N/A = Not applicable

†p value considered significant if < 0.05

^*^p value between children with CM and an abnormal CrCP (below and above 1SD from normal) vs all other CM patients

The normal OP value in pediatric patients, and the presumed OP for our healthy control patients, is 18 ± 10 cmH20 [28]. The mean opening pressure of the cohort of children with CM was not significantly different than this at 18 ± 8 cmH2O (Table 4). There were no significant differences in OP identified between children with CM and a CrCP within 1SD of the age normal compared to those with a CrCP > 1SD below the mean normal (18 ± 6 mmHg) or > 1SD above the mean normal (17 ± 7 mmHg)(*p* = 0.62)(Table 4).

### Physiologic Variables Impacting Vascular Tone

The strongest measurable drivers of vascular tone, blood pH and partial pressure of carbon dioxide (PaCO2), were evaluated. The mean pH in healthy controls was 7.4 ± 0.03 mmHg and the mean PaCO2 was 38 ± 3 mmHg. The mean pH in the cohort of CM patients was 7.39 ± 0.09 mmHg and the mean PaCO2 27 ± 10 mmHg (*p* = 0.9 and *p* = 0.04 compared to controls, respectively, Table 4). However, there were no significant differences in pH or PaCO2 identified between children with CM and CrCP within 1SD of the age normal compared to those with a CrCP > 1SD below the mean normal (7.39 ± 09 mmHg and 27 ± 11 mmHg) or > 1SD above the mean normal (7.4 ± 0.07 mmHg and 24 ± 11 mmHg)(*p* = 0.74 and *p* = 0.35 respectively)(Table 4).

When evaluating other modulators of vascular tone, the mean temperature for the control patients was 36.6 ± 0.3 degrees Celsius, mean arterial blood pressure was 75 ± 8 mmHg, oxygen saturation was 98 ± 1%, and packed cell volume was 34.8 ± 4.2%. Patients with CM had a similar mean temperature of 37.0 ± 0.6 degrees Celsius, mean blood pressure of 74 ± 8 mmHg and median oxygen saturations of 98 ± 2%, but a lower packed cell volume of 24 ± 3% (Table 4). We found no difference in temperature, mean blood pressure, oxygen saturation, or packed cell volume between CM patients with CrCP within 1SD of the normal mean compared to those CrCP > 1SD below the mean normal or above the mean normal (*p* > 0.99 for all, Table 4). Based on EEG, no patient was experiencing subclinical status epilepticus at the time of the TCD examination.

### Venous pressure [29]

In healthy controls, the mean venous flow velocity was 17 ± 1 cm/sec. It was significantly higher in children with CM at 28 ± 11 cm/sec (*p* < 0.001). There were no significant differences in venous flow identified between children with CM and a CrCP within 1SD of age normal (29 ± 9) compared to those with a CrCP > 1SD below age normal (28 ± 10 mmHg) or > 1SD above age normal (28 ± 11 mmHg)(*p* = 0.16).

### DCM

The diastolic closing margin in healthy children was 38 ± 12 mmHg whereas it was 31 ± 14 in children with CM (*p* < 0.001). A low DCM was identified in a total of 39 children with CM (18%), and a critical DCM was identified in 2 children (6%). Patients with a low DCM were significantly older (4.9 ± 2.7 years vs 3.8 ± 2.7 years, *p* = 0.02) and had lower PaCO2 values (22 ± 10 mmHg vs 29 ± 8 mmHg, *p* < 0.0001) than those with a DCM > 20mmHg. Blood pressure adjusted for age, packed cell volume, and opening pressure were not significantly different between children with low DCM compared to the rest of the cohort (*p* > 0.5 for all values, data not shown).

### Outcomes

A good outcome was identified in 155 (70%) children with CM. The rest had a poor outcome, with 30 (14%) being discharged with moderate to severe disability and 35 (16%) dying prior to discharge. CrCP was not associated with outcome. Those children with a good outcome had an average CrCP of 32 ± 12 mmHg whereas those who survived with disability had an average CrCP of 28 ± 14 mmHg and those that died had an average CrCP of 27 ± 15 mmHg (*p* = 0.22).

**Fig. 1:**
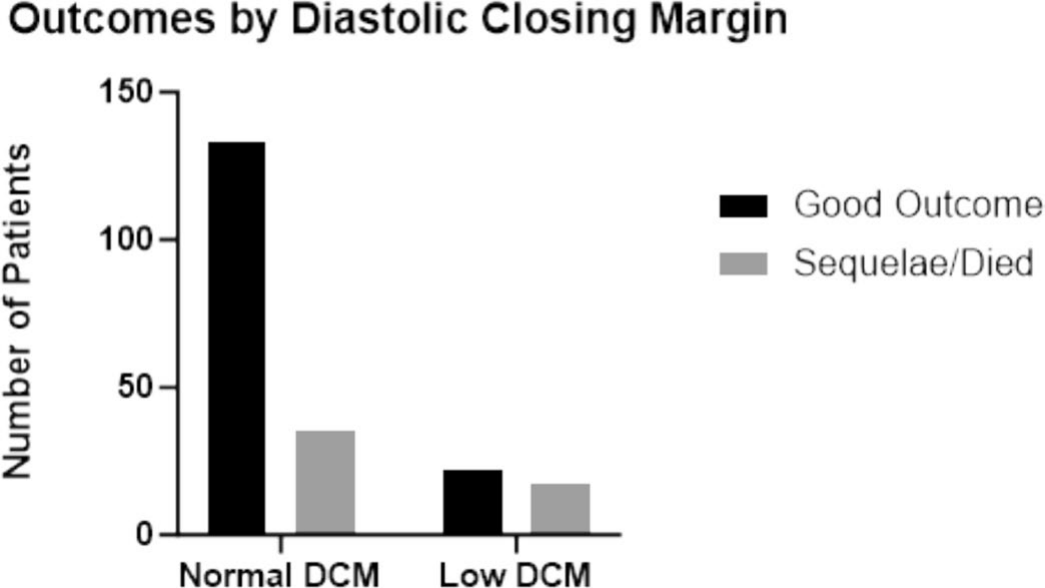
Number of patients with a normal and low diastolic closing margin (< 20 mmHg) with good outcomes vs poor outcomes (sequalae or death)

A normal DCM was seen in 133 (60% of the entire cohort) of children with a good outcome and in 45 (21% of the entire cohort) with a poor outcome. A low DCM was identified in 23 (11%) children with a good outcome and in 19 (8%) with a poor outcome (Fig. 1). A low DCM < 20mmHg conferred a relative risk of poor outcome (death or disability) of 1.4 (95% CI 1.2–1.9)(*p* = 0.008). Both children with a critically low DCM died.

## Discussion

Cerebral malaria impacts thousands of African children annually and is associated with high mortality rates. An improved understanding of the basic pathologic mechanisms of disease is required to rationalize the development and deployment of targeted adjunctive therapies that may improve outcomes. Transcranial doppler ultrasound has identified five distinct alterations to measured CBFVs and morphologic waveforms in two different populations of African children, each associated with different odds ratios of poor outcomes [4, 5]. Further investigations into mechanistic contributors of aberrant cerebral blood flow in CM is paramount as, once identified, these factors may be a target of therapeutic approaches. Previous work has led us to hypothesize that an alteration to vascular tone may be a primary contributor to previously reported deranged CBFVs in some patients with CM. We therefore measured CrCP (CrCP = ICP + vascular tone + venous pressure) to estimate vascular tone in a cohort of children with the disease. Our key findings were 1) Compared to the CrCP in healthy control patients, the CrCP was both significantly lower and higher in many children with CM (17% of children with CM had a CrCP that was > 1SD below the mean CrCP of healthy children and 19% of children with CM had a CrCP that was > 1SD above the mean CrCP of healthy children) 2) A low diastolic closing margin < 20mmHg was associated with increased rates of poor outcome.

### Pathophysiology of Altered CrCP in CM

#### Intracranial Pressure

Critical closing pressure is calculated as: ICP + vascular tone + venous pressure. In CM patients, brain volume scores were significantly higher than is seen in normal individuals. However, as a scoring system of the MRI *appearance* of cerebral hemispheres, increases, particularly to the lower range of the score (i.e. 5–6 as was seen in most patients in this study) may not translate to elevated intracranial pressure given the compensatory mechanisms of the intracranial space. Indeed, in our cohort, ICP, estimated by opening pressure, was not significantly different than published normative pediatric values. Additionally, when evaluating both BVS and OP with CM by different CrCP groupings (within 1SD of age normal value, > 1SD below age normal value, > 1SD above age normal) there were no significant differences identified. This makes increased ICP less likely to be a significant contributor to abnormal CrCP values in many children with CM.

#### Vascular Tone

**Fig. 2:**
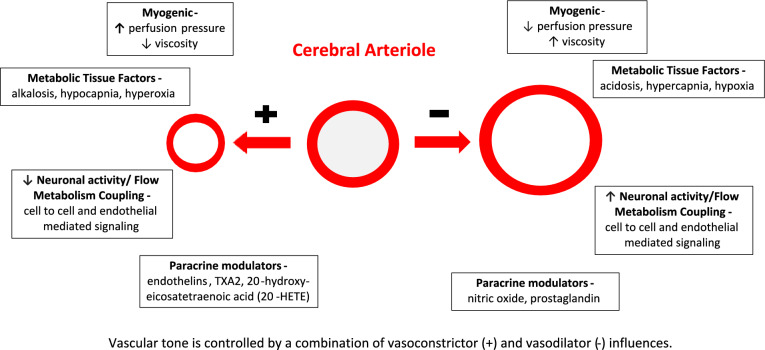
The multiplicity of components that crosstalk to alter vascular tone and maintain brain homeostasis

Beyond ICP, the other most relevant factor contributing to the measured CrCP is vascular tone. Overall, 36% of the cohort with CM had CrCP significantly below or above the CrCP in healthy children. This suggests that derangements to vascular tone (both low and high tone) occur in many children with CM. Vascular tone is controlled by a multiplicity of components that cross talk in attempt to maintain brain homeostasis in response to changing cerebral metabolic demand as well as throughout a variety of pathophysiologic conditions (Fig. 2). The main factors include:

1. Myogenic properties of the blood vessel that are pressure/sheer wall stress dependent
2. Local tissue factors (i.e. pH, PaCO2, O2)
3. Neuronal activity/altered metabolic demand
4. Parenchymal or endothelially derived paracrine modulators/vasoactive peptides (i.e. nitric oxide (NO), endothelin-1 (ET-1), prostaglandin E2 (PGE2), thromboxane A2 (TXA2))

#### Myogenic Factors

Two key myogenic factors that contribute to vascular tone include blood pressure and blood viscosity. In this study, patients with CM had an equivalent mean blood pressure to control patients. There were also no significant differences in the blood pressures of children with CM when evaluating by CrCP grouping. Packed cell volume (equivalent to hematocrit) is a significant contributor to blood viscosity and therefore wall tension. During anemia, blood viscosity decreases, decrease shear force is applied to the cerebrovascular endothelium, and vasoconstriction occurs. While children with CM were significantly more anemic than control patients, it has been repeatedly demonstrated that counter balancing factors including reduced red cell deformability, cytoadherence of infected red cells, and high levels of paraproteins including fibrinogen result in a whole blood viscosity that does not significantly change in severe malarial disease [30–32]. Also, there were no significant differences in PCV when evaluating CM patients by CrCP grouping. Overall, these findings decrease the likelihood that differences in measured CrCP in children with CM occurred secondarily to myogenic factors.

#### Local Tissue Factors

Blood pH and PaCO2 independently and concomitantly regulate cerebrovascular tone [33]. Blood pH was not significantly different between control patients and those with CM nor between CrCP groupings of CM patients. PaCO2 was significantly *lower* in children with CM compared to controls, thus making this a possible contributor to increased cerebrovascular tone in CM, particularly in those with high CrCP. However, if PaCO2 were the only driver of the vascular tone alterations leading to different measured CrCP, those with low CrCP would have been expected to have *normal or significantly higher* PaCO2 than those children with normal or high CrCP. PaCO2 values were not different between children with CM and different CrCPs (less than, within, or greater than 1SD from age normal). Thus, PaCO2 may be contributing to abnormal vascular tone in some individuals, but other factors should also be considered.

Oxygen saturation values were not different between control and CM patients nor between CrCP groupings, making it less likely have contributed to measured CrCP. However, oxygenation saturation was measured peripherally only, and it remains possible that local cerebral tissue hypoxia due to poor delivery or impaired utilization could have contributed to vascular tone derangements in some patients. Direct invasive measurement of brain tissue oxygenation is not possible in our setting, so this could not be measured.

Together, it is less likely that tissue factors such as pH, PaCO2, and oxygen saturation were primary drivers of vascular tone differences between CrCP groupings, albeit the PaCO2 may have influenced tone in some children with high CrCP.

#### Neuronal Activity/Altered Metabolic Demand

The degree of fever was not significantly different between control and CM participants nor between CrCP groupings of CM patients. Additionally, no patient had evidence of subclinical status epilepticus on EEG done concurrently with the TCD/blood pressure measurements. It is therefore unlikely that neuronal activity/flow metabolism coupling contributed to the CrCP differences in CM patients.

#### Paracrine Modulators/Vasoactive Peptides

Given that other contributors to vascular tone are generally unlikely to have contributed to all alterations to measured CrCP in CM patients, deranged levels of parenchymal or endothelially derived vasodilators or vasoconstrictors may be present in the disease, alter cerebrovascular tone, and contribute to the CrCP findings reported. Within the bloodstream of malaria-infected hosts, abnormal levels of various metabolites known to be paracrine modulators have been described (including NO and its precursor L-Arginine, ET-1, bradykinin, kynurenine). However, the relationship between these derangements and altered cerebral blood flow or vascular tone has not been evaluated [34–47]. Future work should investigate the association between the values of these peptides and TCD CBFVs or the CrCP/vascular tone. If found to be associated, future trials could investigate the impact of therapeutic strategies targeting these vasoactive peptides or their precursors.

#### Venous Flow

In the setting of venous occlusion, alternate veins are used for collateral venous drainage, thus increasing flow velocity in these patent vessels [48–51]. In many studies of CM, significant occlusion of small cerebral veins by sequestration of parasitized red blood cells has been reported [52, 53]. Therefore, it is not surprising that venous flow velocities were significantly higher in all children with CM compared to healthy controls. Notable, however, is that high venous flow results in a higher calculated CrCP. This means that the 17% of the CM cohort with CrCP > 1 SD *below* the value in normal children as well as the 64% with CrCP *within* 1 SD from the value in normal children likely had lower vasomotor tone than their calculated CrCP suggests.

#### DCM and its Relationship to Outcomes

Impaired cerebral perfusion is an important contributor to poor outcomes in various forms of acquired brain injury [22]. An effective perfusion pressure requires that the mean blood pressure exceeds the sum of the downstream pressures or the CrCP. When this does not occur, cerebral circulatory arrest occurs. The diastolic closing margin represents the reserve of cerebral perfusion before the cessation of cerebral flow occurs during diastole. We found that a diastolic closing margin < 20mmHg was associated with higher rates of morbidity and mortality. It is likely that the children found to have low DCM at the time of admission had other periods, either pre- or post-hospitalization, where that safety margin was exceeded, leading to minimal or no cerebral perfusion and secondary brain injury. Future work may consider monitoring serial DCM in patients with CM to allow for improved individualization of cerebral perfusion pressure targets that may optimize neuroprotection and improve outcomes in this population. Of note, the reason for low PaCO2 identified in CM patients is unclear and may be multifactorial (neurologically mediated, pulmonary stretch from parasitized red cells in the capillaries, other). However, if the contributor can be identified, it could also be a therapeutic target give the association with low PaCO2 and low DCM.

## Limitations

The most significant limitation of our work is the method by which the CrCP was calculated. The most accurate methods rely on continuous, simultaneous measurements of middle cerebral artery flow velocity and invasive arterial blood pressure [10, 14]. Offline analysis then allows for the correct compensation of the time delay between blood pressure and cerebral flow velocity curves, eliminating hysteresis of the blood pressure/flow velocity plots during regression analysis. Invasive, continuous arterial blood pressure monitoring is not available at our center, nor in most regions where malaria infection is endemic, making this approach not feasible for use in children with CM. We performed simultaneous measurements of both the TCD and blood pressure at the bedside and then took the averaged absolute values to run a linear regression, where CrCP was determined to be the intersection of that line with the x axis. Consequently, this approach gives only a rough estimate of the CrCP. However, given the relatively rapid heart rate of pediatric patients (mean HR 140 in our cohort), the time resolution may have been marginal and our estimates of CrCP reasonable. This is supported by the fact that the mean CrCP of our healthy control patients calculated in this manner was 31 (± 4) mmHg and values of CrCP reported in healthy adults using invasive monitoring and more advanced processing methods are typically around 30 mmHg as well [10]. Future studies in other settings where advanced neuromonitoring of pediatric patients is possible could be done to validate our approach compared to the gold standard of continuous monitoring with offline analysis described above. If validated, our approach has proven feasible to perform and useful to inform immediate clinical decisions in this resource limited malaria endemic setting, which could potentially be extrapolated to allow real time decision making in other settings as well.

Another major limitation of the study is that invasive ICP measurements were not available at our center. Thus, of the three variables that contribute to the calculated CrCP, we could only directly measure one (venous pressure) and had to estimate ICP based on opening pressure in order to infer the vascular tone. Despite not seeing significant differences in opening pressure between groups of children with CM (those with CrCP within 1SD of age normal value, > 1SD below age normal value, > 1SD above age normal), it remains possible that, if ICP had been measured invasively, it would have significantly contributed to CrCP results across the cohort or on an individual patient level. Future studies may assess this in other settings where ICP monitoring is possible. Overall, this limitation makes it impossible for us to firmly conclude that abnormal vascular tone is the definitive driving factor for altered cerebral hemodynamics in children with CM.

## Conclusions

Many pediatric patients with CM have a critical closing pressure below or above normative values. Altered vascular tone may be a significant contributor to these findings. Future studies identifying causative factors for abnormal vascular tone, such as PaCO2 and/or deranged levels of paracrine modulators, could lead to the administration of pathophysiologically relevant adjunctive therapies in this patient population. Additionally, a low diastolic closing margin is associated with worse outcomes, and thus may represent a therapeutic target for future studies.

## Supplementary Information

Below is the link to the electronic supplementary material.

Supplementary file1 (DOCX 189 KB)
