# Supplementary figures and images for: Advancing Understanding of Cerebrovascular Hemodynamic Perturbations in Pediatric Cerebral Malaria Using a Modified Critical Closing Pressure Evaluation- A Prospective, Observational Study
Source: Neurocrit Care. 2025 Apr 21;43(2):493–503. doi: 10.1007/s12028-025-02245-w (PMC12436552; doi:10.1007/s12028-025-02245-w)

**Supplementary Figure 1.**


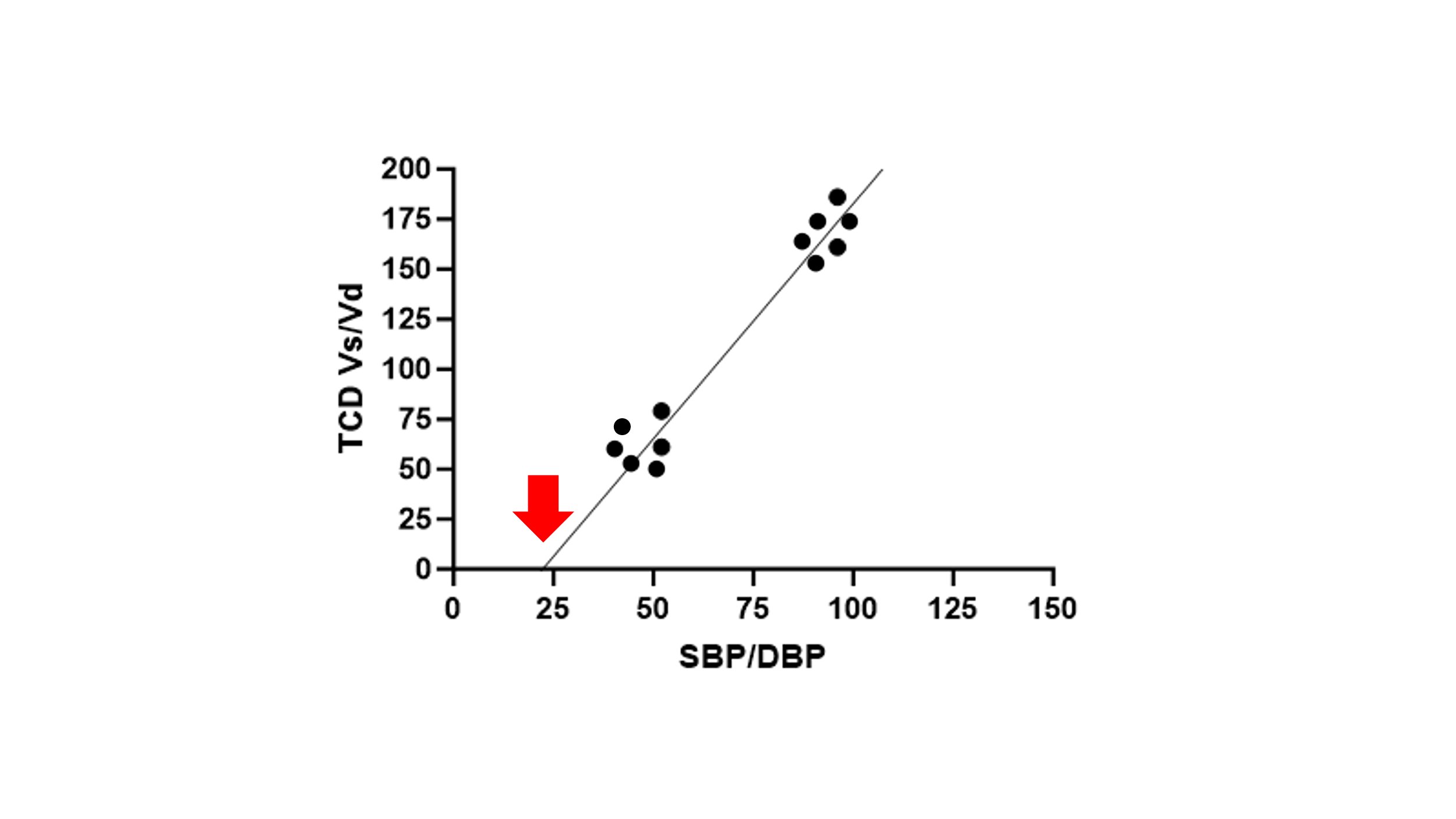

Supplement: Supplementary file 1 — Supplementary file1 (DOCX 189 KB) [file 12028_2025_2245_MOESM1_ESM.docx]
